# Geographical and Ecological Differences in Pepper Cultivation and Consumption in China

**DOI:** 10.3389/fnut.2021.718517

**Published:** 2021-10-12

**Authors:** Ziying Zou, Xuexiao Zou

**Affiliations:** ^1^College of Geographic Science, Hunan Normal University, Changsha, China; ^2^Engineering Research Center of Education Ministry for Germplasm Innovation and Breeding New Varieties of Horticultural Crops, Hunan Agricultural University, Changsha, China

**Keywords:** pepper (*Capsicum*), consumption characteristics, production division, ecological difference, China

## Abstract

Peppers (*Capsicum spp*.) are used as food items, and particularly condiments, across most of the world. Accordingly, these vegetables occupy the largest annual stable planting area (>21,000 km^2^) in China. However, pepper growth, cultivation systems, yield formation, and cultivar traits vary among different environments. China is characteristic for its widely diverse terrains and high ecological heterogeneity, which determine its unique pepper consumption habits and cultivation patterns. The present study provides a comprehensive overview and analysis of the geographical and ecological characteristics of Chinese pepper consumption habits and cultivation systems, and the influence of climatic and human factors on the national pepper planting industry. For this, we analyzed detailed geospatial datasets and reviewed relevant policy papers and academic literature. Based on those findings, we then proposed sustainable management strategies for China's pepper industry; we offered suggestions for aligning the continued development of pepper cultivation with the national objective of achieving an ecological civilization and the nutritional requirements of an increasingly affluent and diverse population.

## Introduction

Food production is an ecosystem service that is vital for human survival (1, 2) and conventional agriculture has allowed the augmentation of this production; however, it affects ecosystem-regulating and -supporting services, such as climate and water regulation, biodiversity, and soil conservation (3–5). Maintaining and improving ecosystem services while simultaneously meeting the food requirements of growing populations are critical challenges for China and the world (6).

Vegetables represent basic food sources that provide vitamins, dietary fiber, and minerals essential for human health. The total vegetable planting area in China has increased from 9.75% in 2000 to 12.57% in 2019 when it reached 20.86 million ha, and the total annual vegetable yield reached 720 million tons (7), which was second only to the total annual grain yield (8). Currently, China's major vegetable crops include Chinese cabbage, tomato, cucumber, cabbage, pepper, eggplant, cauliflower, and broccoli (9). Moreover, ~100 million laborers are engaged in vegetable production in China and the annual value of the vegetable industry output has reached two trillion CNY. Hence, this represents a fundamental industry in the agricultural and rural areas of China, as it is important to ensure food security, maintain laborer employment, expand international trade, and stabilize farmer income (10). Indeed, its rapid development and growing importance, evolution patterns, international commerce, mechanization, and industrial subsidies (11–16) have attracted the attention of researchers. Most studies have focused on national and regional aspects of the vegetable industry and have either discussed industrial achievements and related issues or only addressed a specific component of the industry. However, the sustainable development of China's vegetable industry merit attention, considering its developmental goals related to achieving national food security and an ecological civilization.

Pepper (*Capsicum spp*.) originated in the tropical and subtropical regions of Central and South America and is one of the oldest crops cultivated by humans (17–20). Current research on pepper is focused on its domestication, specialization, evolution, and characteristics via genome sequencing (21–24). The use of pepper-derived products (25), as well as the history, taxonomy, and distribution of the pepper varieties of Indonesia (26) have also been investigated. Based on available historical evidence, pepper was introduced to China in the late 16th century and almost immediately affected the lives of the Chinese population (17, 27, 28). After more than 400 years of development, pepper has become an important vegetable and condiment that plays a major role in maintaining the annual vegetable supply for urban and rural residents (29). Furthermore, it has become the most widely planted vegetable and the most heavily consumed spicy condiment in China.

In this study, we performed a systematic overview of China's pepper industry and explored its historical and modern conditions. Moreover, we examined the geographical and ecological differences of pepper crops across the country to understand the impact of climatic and human factors on these criteria by consulting relevant industry data, policy reports, academic literature, and detailed geographic and meteorological spatial datasets. Finally, we discussed strategies for optimizing the pepper industry management. We hope to lay a foundation for further research on this topic and for the implementation of policies that align the national goals of building an ecological civilization and the sustainable development of the massive pepper industry, as well as the agricultural sector overall.

## Data Acquisition

Data on the pepper industry of China were derived from the National Industrial System of Specialty Vegetables (NISSV, one of the Technological Systems of Modern Agricultural Industry jointly established by the Ministry of Agriculture and the Ministry of Finance of P. R. China). (i) The field data were gleaned from annual statistics in various regions of China. (ii) Data on the spiciness levels of pepper were compiled from multiple questionnaires, surveys, and expert ratings conducted nationwide. (iii) Pepper consumption statistics were acquired from sampling surveys conducted at experimental stations within the NISSV. Meteorological data were extracted from the “dataset of daily surface observation values in individual years (1981–2010) in China” and the “dataset of daily climate data from Chinese surface stations for global exchange (v. 3.0)” from the China Meteorological Data Service Center (30, 31). The latter includes data from basic, reference, and ordinary surface meteorological stations in China, as well as normal daily values for several climatic factors, including temperature, precipitation, relative humidity, sunshine hours. According to numerous literature sources, such as local chronicles and agricultural yearbooks for various provinces in China, a survey was performed to examine the distribution of pepper cultivation across the country. Notably, the national-scale results obtained in this study are not applicable to Taiwan, Hong Kong, and Macau due to limitations in data collection.

## Geographical and Ecological Differences in China's Pepper Cultivation

### History of Pepper Distribution Across China

**Figure 1:**
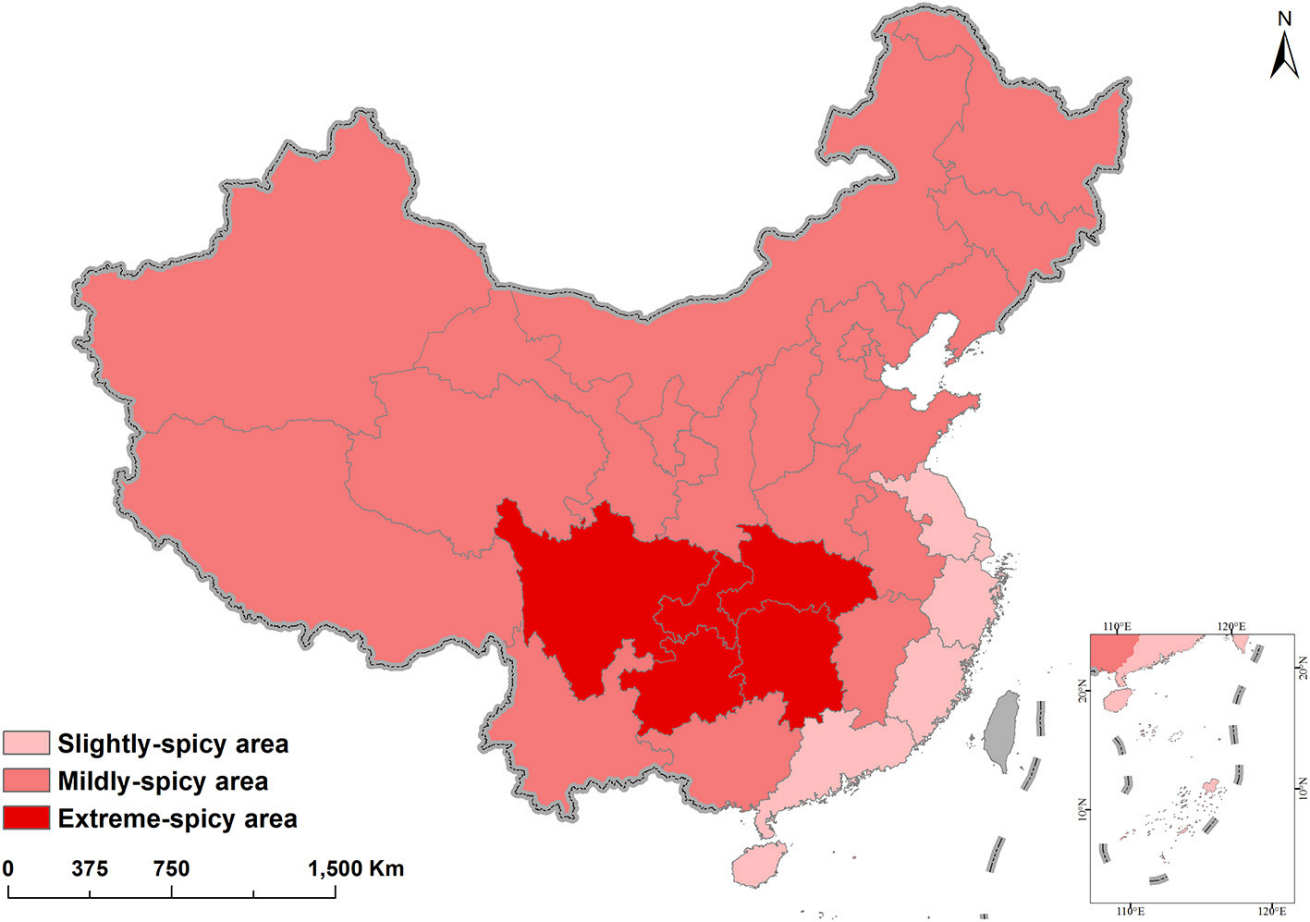
Spatial distribution of pepper spiciness level in China. Data from China's Taiwan, Hong Kong, and Macau are not available.

Pepper was first introduced to Zhejiang Province in 1591, where local chronicles and other historical records show that its earliest name was “Fan-pepper.” It was then regionally distributed to the south, west, and north of the country, originating a pepper consumption with Chinese characteristics (32). Although Jiangsu Province did not accept this new type of pepper, the crop was received in Shandong Province, where it was quickly accepted as an alternative to the Chinese pepper (genus *Zanthoxylum*) and consequently spread to other provinces in northern China, where it became known as “Qin-pepper” (33, 34) (with the exception of Shanxi, Inner Mongolia, Xinjiang, and Qinghai). Ultimately, regions favoring mildly spicy pepper were established, including Beijing, Tianjin, Shandong, Hebei, Shanxi, Henan, Inner Mongolia, Liaoning, Jilin, Heilongjiang, Shaanxi, Gansu, Ningxia, Qinghai, and Xinjiang (Figure 1).

Pepper then spread through southern China. Fujian, Guangdong, and southern Guangxi did not accept this new type of pepper, while Jiangsu, Shanghai, and Zhejiang became known as the regions of the southeastern coast of China with slightly spicy pepper. All coastal areas except Guangxi referred to it as “Fan-pepper,” which is not to be confused with the name used for the indigenous species. As the earliest recorded name for this vegetable in Guangxi was simply “pepper”, as opposed to “Fan-pepper”, the pepper of Guangxi was probably received from Hunan rather than Guangdong.

Later, pepper spread across western China along the Yangtze River. Anhui, Jiangxi, and Hubei did not initially accept this new kind of pepper because of the lifestyle habits of its residents. After pepper arrived in Hunan, the locals accepted it and used it as a condiment and vegetable. Pepper became a daily food item that was indispensable for the residents of Hunan, which went on to become the province with the largest pepper consumption in China. The use of pepper as a vegetable and a condiment has since spread to neighboring provinces and cities. Guizhou, Sichuan, Yunnan, Guangdong, Jiangxi, Hubei, and Guangxi all import pepper from Hunan, which has become China's largest pepper distributor. It supplies the areas of the middle and upper reaches of the Yangtze River where extremely spicy pepper is preferred, including Hubei, Jiangxi, Guizhou, Yunnan, Sichuan, Chongqing, and southern Shaanxi.

### Pepper Varieties and Industrial Production Scale in China

Pepper is a thermophilic vegetable that is insensitive to the photoperiod but sensitive to effective accumulated temperature. As such, it can be planted in most regions, including Tibet in the west, Shanghai in the east, Hainan in the south, and Heilongjiang in the north. Over 95% of the crops produced in China are *Capsicum annuum* L., *Capsicum frutescens* L., and *Capsicum chinense* Jacq, which are grown partially in Hainan and southern Yunnan. An important variety is Yunnan millet pepper is an important variety of *C. frutescens* L. and *C. chinense* Jacq is the famous yellow lantern pepper of Hainan. As China strongly encourages scientific research on pepper, numerous researchers are interested on its breeding characteristics. Each year, almost 100 new pepper varieties meeting the needs of different markets are selected for production; green, high-efficiency production technologies have ensured that the needs of the market are met in different seasons (early spring, late autumn, and tropical winter) and under various cultivation conditions (e.g., high altitude). Thusly, a balanced supply of fresh peppers has been made available in China.

Based on the statistical data of the National Industrial System of Bulk Vegetables, the total pepper planting area reached 21,474 km^2^ in 2019 and surpassed those of Chinese cabbage and tomatoes. Thus, pepper now occupies the largest vegetable planting area in China (Table 1).

**Table 1:** Annual planting area of the 20 major vegetable crops in China, as of 2019.

**Vegetable species**	**Planting area (km^**2**^)**	**Vegetable species**	**Planting area (km^**2**^)**
Pepper	21,474	Celery	6,773
Chinese cabbage	18,625	Cowpea	6,326
Tomato	14,047	Scallion	5,883
Cabbage	13,531	Spinach	5,833
Cucumber	12,758	Lettuce	5,542
Radish	11,291	Carrot	4,417
Garlic	9,249	Chinese chive	4,238

**Figure 2:**
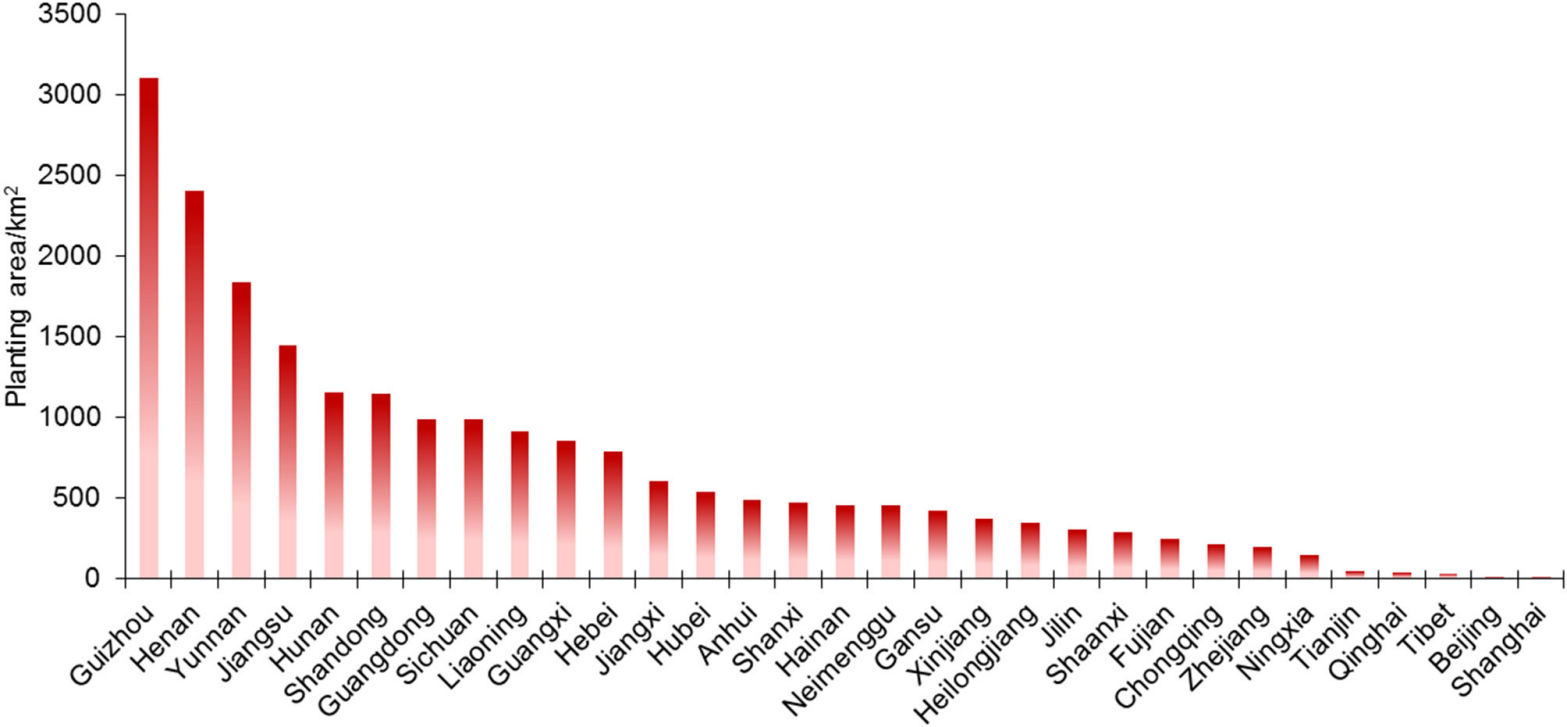
Pepper planting area of each province of China.

**Figure 3:**
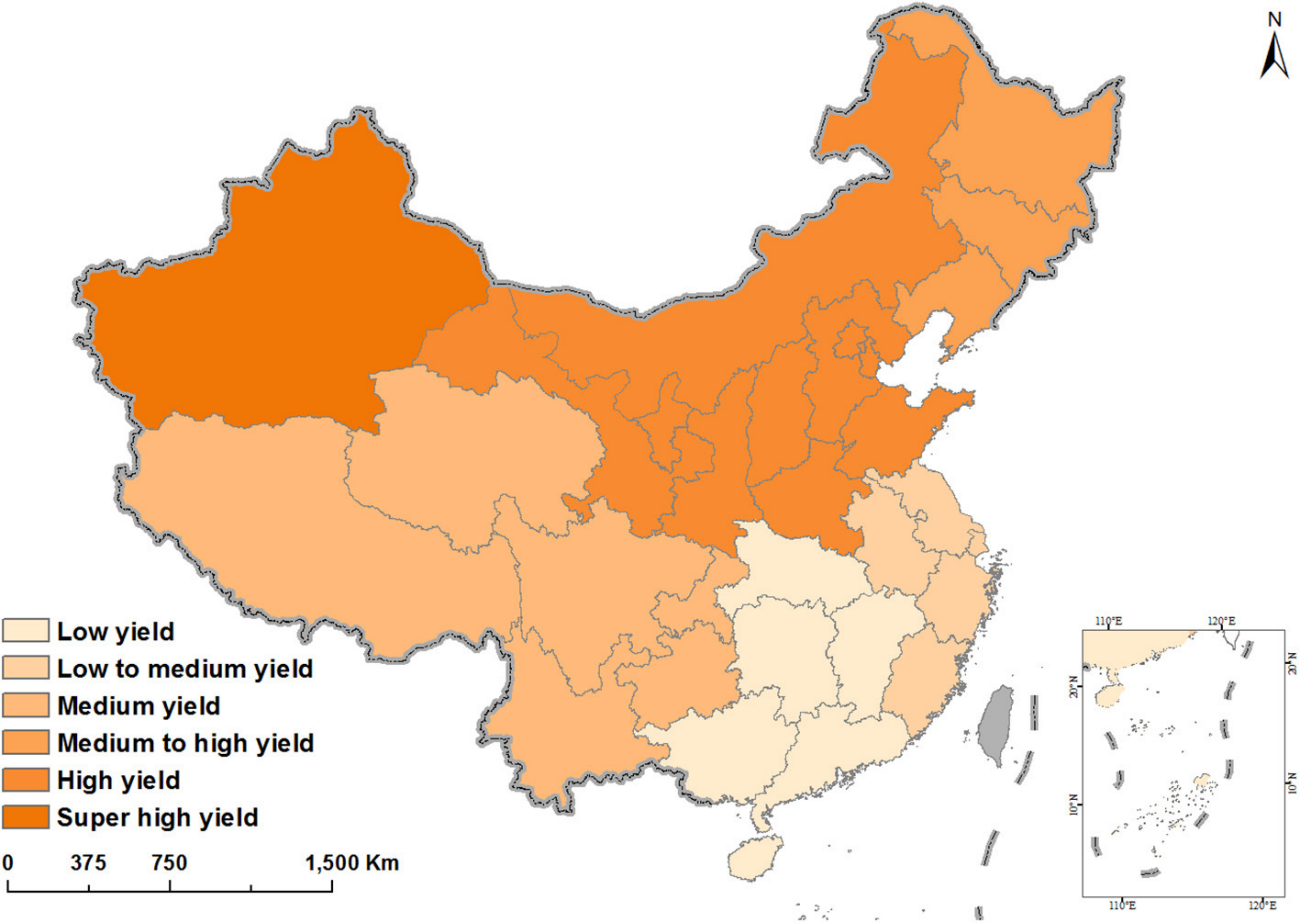
Distribution of pepper yield in China. Data from China's Taiwan, Hong Kong, and Macau are not available.

The total pepper yield in 2019 exceeded 64 million tons and accounted for 7.76% of the total national vegetable output, with the average yield reaching nearly 2,000 kg/mu. The value of the total pepper yield of China reached 250 billion CNY, accounted for 11.36% of the total national vegetable output value, and contributed to 1.14% of the total farm income. Currently, the largest pepper planting area is located in Guizhou with >3,070 km^2^, followed by Henan and Yunnan, with a total pepper planting area of >1,500 km^2^. In the Shandong, Hunan, and Jiangsu provinces, the planting area exceeds 1,000 km^2^ (Figure 2). The changing trend in the pepper industry pattern indicates that production is concentrated mainly in the main cultivation areas. Facility pepper cultivation has rapidly developed in China and its area accounts for 26.0% of that for total national pepper cultivation (Figure 3).

### Ecological Differences in the Distribution of Pepper Cultivation in China

To study the ecological impact of the distribution of pepper production in China, we compared the environmental factors of different regions, such as the length of pepper growth (d) and development period in the temperature range of 20–25°C (including daily average and maximum temperatures), daily average temperature (°C), average temperature difference between daytime and nighttime (°C), relative humidity (%), and annual rainfall (mm). There are obvious differences in climate among regions, which can considerably affect pepper yield (Table 2). Northern China has high pepper yields and is characterized by cool summers with large temperature differences between day and night, low rainfall, and low relative humidity. Moreover, the daily maximum temperature is often 20–25°C during pepper flowering and fruiting. Central, eastern, and southern China have low pepper yield and are characterized by hot summers (maximum temperature >35°C) with extended periods of high temperatures and frequent abnormal flowering and fruiting for extended periods. The low yield is caused by the small temperature differences between day and night, which are not conducive to nutrient accumulation during pepper growth. These areas are also characterized by high rainfall and humidity, which conducive to the proliferation of microbial pathogens in pepper.

**Table 2:** Differences in pepper flowering and fruiting periods under regions with different climates.

**Region**	**Province**	**Cultivation season**	**FFT (month)**	**D1 (d)**	**D2 (d)**	**ADT (^**°**^C)**	**ATD (^**°**^C)**	**ARH (%)**	**R (mm)**	**High yield index**
Northeast China	Heilongjiang	Spring	Jun–Sept	60	37	18.7	10.9	69.7	413.4	5
	Jilin	Spring	Jun–Sept	71	33	19.6	10.8	68.1	469.8	6
	Liaoning	Spring	May–Sept	91	49	20.6	10.1	72.7	527.0	7
	#Average	-	-	74	39.7	19.6	10.6	70.2	470.1	6.0
Northern China	Beijing	Spring	May–Sept	73	20	22.9	11	60.8	454.9	8
	Inner Mongolia	Spring	Jun–Sept	60	25	18.8	12.6	50.8	284.6	8
	Tianjin	Spring	May–Sept	66	22	23.5	9.5	64.4	426.4	7
	Hebei	Spring	May–Sept	69	23	22.8	11.2	63.0	465.5	9
	Shandong	Spring	May–Sept	68	22	23.3	9.7	64.5	629.1	8
	Henan	Spring	May–Sept	60	17	24.1	9.9	63.5	492.8	7
	Shanxi	Spring	May–Sept	88	44	20.2	12.4	59.5	368.6	9
	#Average	-	-	69.1	24.7	22.2	10.9	60.9	446.0	8.0
Eastern China	Shanghai	Spring	May–Oct	70	52	23.8	7.0	70.8	759.7	4
	Jiangsu	Spring	May–Oct	68	45	23.1	8.1	72.2	696.8	4
	Zhejiang	Spring	May–Oct	74	37	24.2	8.1	71.1	860.8	2
	Fujian	Spring	May–Oct	79	27	24.9	8.5	71.3	997.5	3
	Anhui	Spring	May–Oct	76	35	23.3	8.7	76.0	694.5	5
	#Average	-	-	73.4	39.2	23.9	8.1	72.3	801.9	3.6
Central China	Hubei	Spring	May–Oct	72	32	23.5	8.6	74.0	839.5	3
	Hunan	Spring	May–Oct	71	31	24.3	8.2	71.0	749.9	1
	Jiangxi	Spring	May–Oct	69	26	24.9	8.6	70.4	853.2	2
	#Average	-	-	70.7	29.7	24.2	8.5	71.8	814.2	2.0
Southwest China	Chongqing	Spring	May–Oct	79	25	23.7	8.1	74.1	794.4	2
	Sichuan	Spring	May–Oct	108	57	20.2	9.0	77.5	768.8	3
	Guizhou	Spring	May – Oct	130	54	21.5	8.1	77.7	793.1	4
	Yunnan	Spring	May–Oct	127	61	20.4	9.0	74.6	744.1	5
	#Average	-	-	111	49.3	21.5	8.6	76.0	775.1	3.5
Southern China	Guangxi	Spring	Apr–Jun	43	14	24.3	7.6	78.9	417.0	2
	Guangdong	Spring	Apr–Jun	36	13	24.9	7.0	80.0	938.7	3
	Hainan	Spring	Apr–Jun	27	0	27.1	8.1	79.4	557.1	2
	#Average	-	-	35.3	9.0	25.4	7.6	79.4	637.6	2.3
Qinghai-Tibet	Tibet	Spring	Jun–Sept	0	47	13.1	12.2	50.7	377.9	5
	Qinghai	Spring	Jun–Sept	0	31	11.7	12.7	66.4	308.1	5
	#Average	-	-	0.0	39.0	12.4	12.5	58.6	343.0	5.0
Northwest China	Shaanxi	Spring	May–Sept	98	45	21.1	11.1	64.8	411.9	8
	Gansu	Spring	May–Sept	24	83	17	12.0	59.0	221.0	7
	Ningxia	Spring	May–Sept	65	65	18.6	12.3	52.1	161.9	7
	Xinjiang	Spring	May–Sept	96	49	20.9	13.9	39.8	155.0	10
	#Average	-	-	70.8	60.5	19.4	12.3	53.9	237.5	8.0
National wide	#Average	-	-	68.3	36.2	21.6	9.8	67.4	568.8	5.2

*FFT is the pepper flowering and fruiting time (month). D1 is the number of days when temperature is at 20–25°C during pepper growth period and calculated from average daily temperature (d). D1 is the number of days at 20–25°C during pepper growth period and calculated from highest daily temperature (d). ADT is the average daily temperature (°C). ATD is the average temperature difference between the daytime and nighttime temperatures (°C). ARH is the average relative humidity (%). R is the average annual rainfall (mm)*.

The analysis of the correlation between high pepper yield and various regional climatic factors demonstrated that the high yield index was positively but not significantly correlated with the number of days during which pepper growth and development occurred at 20–25°C. This metric was calculated using the daily average and maximum temperatures. The high yield index was positively and highly significantly (*p* < 0.01) correlated with the temperature differences between daytime and nighttime. The high yield index was negatively correlated with daily average temperature (*p* < 0.05), relative humidity (*p* < 0.01), and annual rainfall (*p* < 0.01) (Table 3).

**Table 3:** Coefficients of correlation between pepper yield and climatic factors in different ecological regions of China.

**Region**	**D2**	**D1**	**ADT**	**ATD**	**ARH**	**R**
National wide	0.11	0.22	−0.36^*^	0.78^**^	−0.73^**^	−0.71^**^
China North	0.69^**^	−0.06	0.62^*^	0.19	−0.42	−0.10
China South	0.49	0.61^*^	−0.57^*^	0.17	0.10	0.06
Northeast China and Beijing-Tianjin-Hebei	0.27	−0.52	0.82^*^	0.17	−0.65	0.32
Northeast China and Northern China	0.33	−0.18	0.35	0.38	−0.60	−0.07
Northern China	0.66	0.64	−0.48	0.68	−0.23	−0.16
Northwest China	0.90^*^	0.21	0.91^*^	0.34	−0.53	−0.47
Northern China and Northwest China	0.87^**^	−0.08	0.67^*^	0.11	−0.28	−0.01
Eastern China	−0.21	0.28	−0.69	0.05	0.72	−0.72
Central China and Southern China	−0.21	−0.11	−0.16	−0.26	0.45	0.40
Southwest China	0.91	0.83	−0.69	0.45	0.12	−0.69
Central China and Southwest China	0.85^*^	0.82^*^	−0.79^*^	0.48	0.64	−0.22
Central China and Southern China	0.83*	0.59	−0.91^**^	0.86^**^	−0.84^**^	−0.88^**^
Southwest China and Southern China	0.77^*^	0.80^*^	−0.72	0.46	−0.34	0.43
Eastern China and Southern China	0.54	0.63	−0.66	0.16	−0.29	0.18
Southwest China and Northwest China	−0.06	0.34	−0.08	0.79^**^	−0.78^**^	−0.81^**^
Northern China and Central China	0.06	−0.16	−0.60	0.83^**^	−0.78^**^	−0.86^**^
Northern China and Eastern China	−0.13	−0.47	−0.60^*^	0.85*^*^	−0.77^**^	−0.88^**^

** *Significantly correlated at the 0.01 level (two-sided)*.

* *Significantly correlated at the 0.05 level (two-sided)*.

*D1 is the number of days at 20–25 °C during the growth period of pepper as calculated by the average daily temperature (d). D1 is the number of days at 20–25 °C during the growth period of pepper as calculated by the highest daily temperature (d). ADT is the average daily temperature (°C). ATD is the average temperature difference between day and night (°C). ARH is the average relative humidity (%). R is the annual rainfall (mm)*.

We separately analyzed the correlations between high pepper yield and the climatic characteristics of northern and southern China (Table 3). In northern China, there was a significant correlation between the high yield index and the number of days during the pepper growth period when the temperature was 20–25°C (average daily temperature), as well as between the high yield index and the average daily temperature. In southern China, the number of days when the temperature was 20–25°C (highest daily temperature) and the average daily temperature were significantly correlated with the high yield index. However, the correlations between the high yield index and the climatic factors in various northern regions were not evident. In certain regions, significant correlations between the high yield index and both the number of days when the temperature reached 20–25°C (highest daily temperature) and the average daily temperature were found. The correlations between the high yield index and the relative humidity and rainfall were not significant. Moreover, the correlations between the high yield index and the climatic factors in southern China were stronger than those for northern China. Central and eastern China had the strongest correlations among these factors, followed by central and southwestern China. The correlations among these factors in the other regions were weak and not significant. The analysis of the interlaced north–south areas of China revealed that the overall correlations between the high yield index and climatic factors there were significantly stronger than those of northern or southern China. The high yield index was significantly correlated with the temperature difference between daytime and nighttime, relative humidity, and rainfall.

## Geographical Differences in Pepper Consumption in China

### Overview of Pepper Consumption in China

Pepper is mainly consumed as a fresh vegetable and a processed spicy condiment, and pepper accounts for 30.88% of all condiments consumed in China (35). The pepper planting areas are evenly divided (50/50) for the production of fresh and processed peppers.

**Figure 4:**
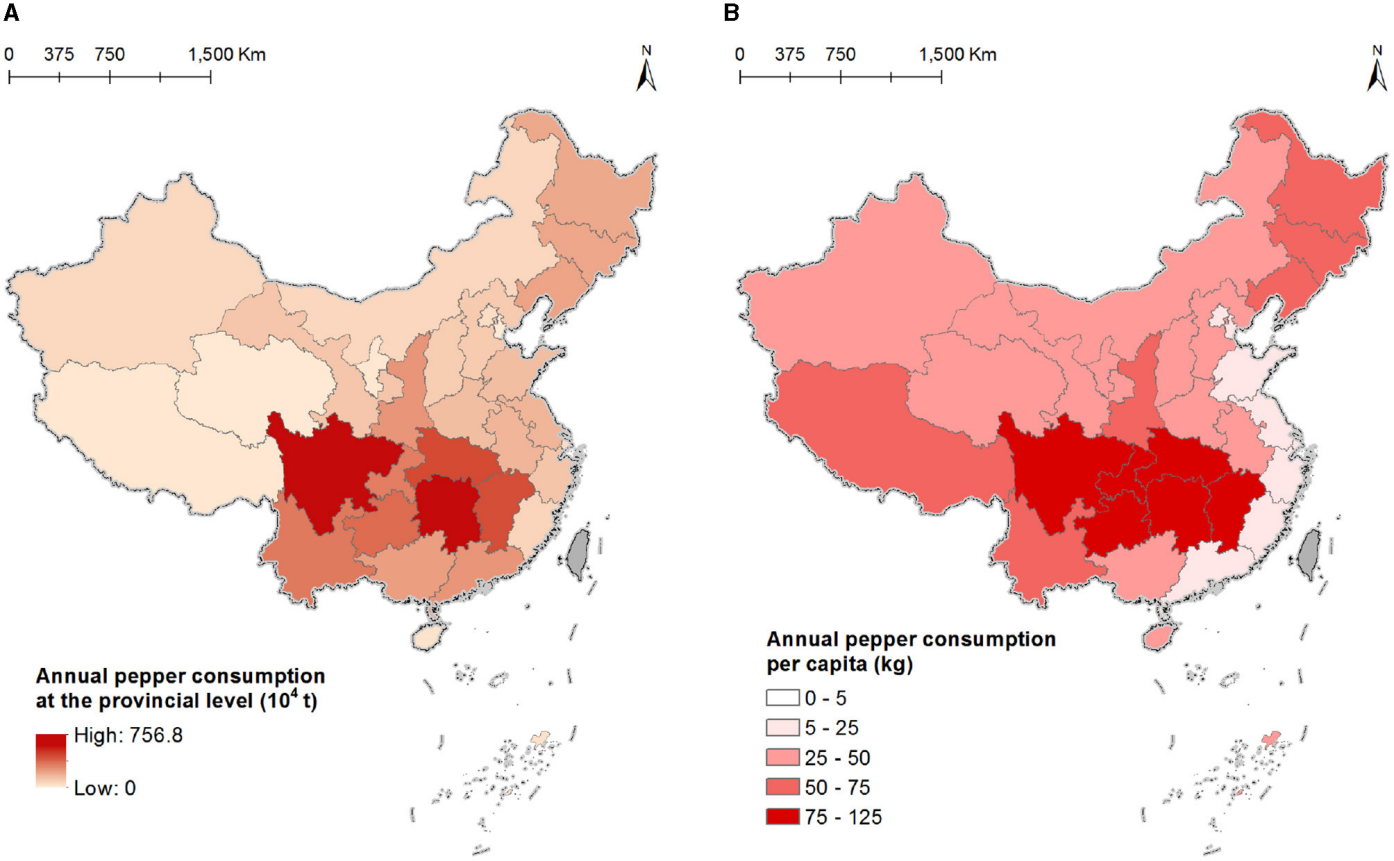
Annual provincial-level pepper consumption rates. Data from China's Taiwan, Hong Kong, and Macau are not available. **(A)** Total pepper consumption in each province. **(B)** Per capita pepper consumption.

Statistical data obtained from the NISSV revealed the distribution of annual pepper consumption in all provinces and includes total and per capita consumption by province (Figure 4). Hunan and Sichuan provinces have the highest total annual pepper consumption (7.57 million tons and 6.04 million tons, respectively), followed by Hubei, Jiangxi, Guizhou, Chongqing, and Yunnan. The total annual consumption per capita reflects the pepper consumption ability of various regions; as such, we calculated the total per capita consumption per province based on the total population at year end. The distribution pattern was consistent with that for the regional pepper consumption preference in various regions. The areas in the upper and middle reaches of the Yangtze River that prefer an extremely spicy diet have the highest per capita pepper consumption (>75 kg), including Hunan Province (111.57 kg), Guizhou (98.5 kg), Jiangxi (97.6 kg), and Chongqing (95.6 kg).

### Climatic Factors Causing Geographical Differences in Pepper Consumption

To clarify the relationships between climatic factors and Chinese pepper consumption, we calculated the average annual sunshine hours (h), daily average temperature (°C), relative humidity (%), annual rainfall days (d), and annual rainfall (mm) in various areas that prefer a spicy diet. The preference for a spicy diet in certain regions of China is related to their weather. The areas that prefer an extremely spicy diet, the weather is characterized by high temperatures and humidity and the occurrence of many cloudy and rainy days and few sunny days and sunshine hours, while those with a preference mildly spicy diets are characterized by lower temperatures, the occurrence of many sunny days and few rainy ones, and abundant sunshine. Moreover, although the weather in areas preferring slightly spicy diets is also characterized by high temperatures and relative humidity, it has more sunshine hours, more cloudy and rainy days and fewer sunny days than the areas that prefer extremely spicy diets.

The comparison of the climatic factors in areas with different spiciness level preferences revealed that those preferring slightly spicy diets have the greatest number of annual sunshine hours, followed by those with a preference for mildly spicy diets, while the areas preferring extremely spicy diets have the fewest annual sunshine hours and the greatest number of annual rainfall days. In contrast, the slightly spicy diet areas have the fewest annual rainfall days, followed by those preferring mildly spicy diets. The average daily temperature and annual rainfall are highest in the areas preferring slightly spicy diets, followed by those that prefer extremely spicy diets, with the areas that prefer mildly spicy diets presenting the lowest values. The relative humidity of the areas with extremely spicy diets is similar to that of those preferring slightly spicy diets, and both are significantly higher than that of the areas with mildly spicy diets.

The correlation analysis between the level of food spiciness and the climatic factors showed that the level of food spiciness in the areas with extremely spicy diets was mainly positively correlated with the number of annual rainfall days and the relative humidity, and negatively correlated with the number of annual sunshine hours (Table 4). The correlation coefficient between the diet spiciness level and the number of annual rainfall days was significant (*p* < 0.05). The level of food spiciness in the areas with mildly spicy diets was positively correlated with the average daily temperature and relative humidity, and negatively correlated with the number of annual sunshine hours. Nevertheless, these correlations were not significant. In the areas with mildly spicy diets, there was a poor correlation between pepper consumption habits and climate, and the former is partially determined by lifestyle habits. In the areas with slightly spicy diets, there was virtually no correlation between pepper consumption habits and climate, and the former is fully determined by lifestyle habits. On a national level, the level of food spiciness was negatively correlated with the number of annual sunshine hours, significantly positively correlated with the number of rainy days (*p* < 0.05), and weakly correlated with the daily average temperature, relative humidity, and annual rainfall. The number of annual rainfall days had the strongest impact on pepper consumption habits. As such, we conclude that local preference for spicy diets increases with rainfall and decreases with increasing number of annual sunshine hours.

**Table 4:** Climatic differences among regions of China with different spiciness level preferences.

**Spicy-diet area**	**Province**		**Annual sunshine hours (h)**	**Average daily temperature (^**°**^C)**	**Average relative humidity (%)**	**Annual rainfall days (d)**	**Annual rainfall (mm)**
Extreme-spicy area	Hunan	10	1,568.7	17.4	72.2	181.9	1,340.1
	Hubei	7	1,765.4	16.4	73.5	124.7	1,198.3
	Jiangxi	8	1,851.3	18.0	70.8	147.3	1,559.9
	Chonqing	9	982.2	17.5	76.3	153.3	1,052.7
	Sichuan	9	957.4	14.7	76.4	156.8	860.8
	Guizhou	9	1,003.6	15.7	78.0	200.8	1,005.7
	Yunnan	7	2,214.3	16.5	67.8	130.4	855.3
	#Average	8.4	1,477.6	16.6	73.6	156.5	1,124.7
	#CC	-	−0.68	0	0.53	0.82*	0.1
Mildly-spicy area	Guangxi	3	1,534.7	20.6	77.7	174.6	1,240.1
	Guangdong	1	1,575.5	21.8	73.7	149.2	1,916.4
	Hainan	2	1,875.5	24.3	80.2	102.5	1,975.9
	Shanghai	1	1,710.1	16.5	69.4	93.7	1,143.5
	Jiangsu	1	1,911.3	15.3	69.7	123.0	1,012.7
	Zhejiang	1	1,875.2	17.2	70.2	151.9	1,416.2
	Fujian	2	1,739.0	19.3	70.8	88.8	1,505.7
	#Average	1.6	1,745.9	19.3	73.1	126.2	1,458.6
	#CC	-	−0.42	0.51	0.69	0.23	0.07
Slightly-spicy area	Anhui	5	2,055.0	15.6	73.3	144.1	1,002.6
	Shandong	4	2,194.2	13.4	56.0	79.2	742.4
	Henan	3	1,841.4	14.7	57.4	85.0	631.4
	Beijing	4	2,453.8	12.0	51.7	66.6	552.2
	Tianjin	4	2,252.1	12.6	57.9	63.8	519.2
	Hebei	4	2,726.5	11.9	55.9	71.3	558.4
	Shanxi	4	2,519.9	9.8	53.8	70.3	455.3
	Inner Mongolia	4	2,734.4	4.9	46.5	70.0	392.9
	Liaoning	4	2,533.1	8.7	67.2	89.3	730.4
	Jilin	4	2,499.8	5.5	61.0	98.7	649.6
	Heilongjiang	4	2,555.7	3.0	63.8	104.2	577.9
	Tibet	5	2,982.5	5.6	35.4	92.1	438.4
	Shaanxi	5	1,819.1	11.8	62.2	90.3	549.7
	Gansu	5	2,502.9	8.1	55.6	18.8	268.7
	Qinghai	4	2,501.1	2.5	56.7	96.9	428.6
	Ningxia	4	2,732.6	8.3	49.2	28.6	205.2
	Xinjiang	4	2,868.1	8.8	55.2	86.9	290.1
	#Average	4.2	2,457.2	9.2	56.4	79.8	529.0
	#CC	-	0.05	−0.04	0	0.09	0.02
National scale	#CC	-	−0.35	−0.01	0.18	0.41*	0.08

#Average: provincial average in each spicy diet area; #CC: correlation coefficient; *significantly correlated at the 0.05 level.

### Effects of Socioeconomic and Cultural Factors on Pepper Consumption

The Chinese spicy diet is related to its climate and local economy. Although high temperatures and humidity and prolonged rainy weather contribute to increased pepper consumption, pepper consumption in areas with suitable climatic and sunshine characteristics is influenced not by the climate but rather by the poor economy. For these regions, we investigated the factors contributing to geographical differences in pepper consumption.

Central China (Hunan, Hubei, and Jiangxi) has high humidity and the local weather is prone to sudden and drastic changes. Hence, the residents of Hunan consume pepper to dispel cold and dampness. Although traffic, political, economic, and cultural conditions have improved in modern times, the region remains economically underdeveloped. Agricultural production is simple and no other crops except rice are planted to any great extent, considering their lower yield and poorer quality. Agricultural products are relatively scarce, and the food structure is relatively simple. As pepper acts a disinfectant, stimulates taste, and is comparatively inexpensive, it is the preferred rice condiment for the residents of Hunan. Jiangxi and Hubei are adjacent to Hunan and have a similar climate, economy, and dietary structure. Therefore, their residents have a similar demand for pepper. This is also the case of southwestern China, whose climate is characterized by rain and fog, high humidity, and relatively little sunshine, but its economy is relatively poor, and life is comparatively primitive. Therefore, its residents also use *Capsicum* pepper to dispel cold and dampness, as this condiment is generally cheaper than other spice ingredients.

Shandong was the first province in China known to consume pepper (22). Today, residents of northwestern, northern, northeastern, and other regions of China consume Chinese pepper (*Zanthoxylum* spp.) as a condiment. *Capsicum* pepper is spicier than Chinese pepper. As the production cost of *Capsicum* pepper is significantly lower than that of Chinese pepper, its consumer cost is relatively low and it has a wide cultivation range. Moreover, the economy is poorly developed in northern China and its residents use pepper as a condiment for rice, making it the staple food in their daily diet. Hence, the consumer demand for pepper persists in these regions. As the climate in these areas is relatively dry, the residents do not use peppers to dispel cold or dampness. Consequently, their demand for pepper and requirement for spiciness are moderate.

Pepper was first introduced to China from the coastal areas, which now constitute part of the typical “world pepper belt” as their climatic conditions are conducive to pepper consumption. However, in reality, the residents of the coastal areas of China seldom consume pepper because these regions are relatively affluent and have a wide variety of foods and condiments on offer. Therefore, people in the coastal areas of China need not rely on *Capsicum* or Chinese pepper as a condiment. Moreover, the residents in these areas prefer food that is slightly seasoned, rather than very spicy food.

## Discussion

The foregoing analyses show that, in areas with high pepper yield such as northern China, the climate is characterized by cool weather, low rainfall, sufficient sunshine, wide temperature differences between daytime and nighttime, and dry air. Additionally, temperature has the strongest impact on pepper yield, while humidity has an almost negligible effect. This is mainly because pepper originated in the semi-arid high mountains of Bolivia. Although pepper is thermophilic, it does not tolerate excessively high temperatures. Moreover, although it prefers dryness, it tolerates neither drought nor high humidity. Pepper is highly susceptible to microbial pathogenesis at high temperatures and relative humidity. In China, pepper is cultivated in areas with low rainfall and on irrigated arable land to meet its water requirements. Therefore, temperature is a key limiting factor in pepper cultivation. In areas with low pepper yield such as southern China, the summertime temperatures far exceed the optimal flowering and fruiting temperature of peppers and reduce net crop yield. In addition, the prolonged heavy rainfall causes serious disease in pepper which, in turn, lowers the yield. Hence, temperature and humidity strongly influence pepper production.

Furthermore, there is an imbalanced development of imports and exports in major production and consumption provinces. Comprehensive comparisons of pepper production and consumption in various provinces of China revealed that the areas of Hunan, Sichuan, Jiangxi, Hubei, and Chongqing with extremely spicy diets produce only moderate amounts of the crop as the climate conditions of these regions are not conducive to large-scale pepper production. However, pepper consumption substantially surpasses pepper production in these areas and thus, pepper must be imported from other regions. In fact, each province in this region imports over two million tons of pepper per year; for instance, Hunan Province imported 4.023 million tons of pepper in 2019. In contrast, pepper production considerably outstrips pepper consumption in Henan, Shandong, Liaoning, and other provinces in that region where large quantities of pepper are exported.

The planting area of pepper in China increased from 200 km^2^ in the 1930s to 800 km^2^ in the 1960s, and then increased from 2,700 km^2^ in the 1980s to 13,500 km^2^ by 2000 (36). However, China's pepper industry still faces some issues. Firstly, although pepper breeding has basically met production needs, there is still a gap with market demand. Secondly, compared with the United States and Mexico, the output of pepper per unit area in China is relatively low (37). Finally, China's large-scale pepper industry is relatively low, and it is difficult to promote standardized cultivation techniques and mechanized production. These limitations have restricted the improvement of the output and quality of the pepper industry. At the time of the present study, the planting area has stabilized at 21,350 km^2^. The analysis of the effects of regional climate and geographic differences on pepper consumption, cultivation systems, and yield provides a scientific basis for optimizing the pepper industry in China. We hereby propose the following strategies and measures for the development of the pepper industry in China.

1. Pepper products should be processed as fast as possible in competitive production areas. With the rapid development of the Chinese economy, the costs of transportation of pepper products should be greatly reduced (38). The cost of pepper production can be further reduced if production near consumption areas is rapidly changed to concentrated production in competitive production areas.
2. The patterns of pepper production should be segregated by end use commodity type. Pepper is both a widely consumed spice and condiment and an important green vegetable. It is crucial that a balanced supply of fresh peppers be maintained throughout the year (39). As the territory of China is vast, its latitude and altitude spans are very wide and thus, pepper production methods and market times vary dramatically among producing areas. Pepper production must be planned in accordance with the ecological characteristics of each producing area. Accurately forecasting the market time will help ensure that fresh peppers are supplied every year.
3. Facility cultivation of pepper must also be developed. In the hot and rainy southern regions, pepper growth is improved by cultivation in early spring, late autumn, and under rain protection. In this manner, the adverse effects of unfavorable weather conditions can be overcome, and the market time is altered such that the economic benefits of pepper planting are enhanced. In cold northern high-latitude, high-altitude areas with sufficient sunlight, greenhouses can overcome the insufficient effective accumulated temperature, extend pepper fruiting time, and increase crop yield.
4. Farmland water storage facilities must be improved in competitive pepper production areas. The high yield areas of pepper are distributed in the cold and arid regions of northern and northwestern China. Hence, irrigation is essential for satisfactory pepper production (40); however, drip fertigation should be applied wherever feasible.
5. Pepper processing should also be developed. Processing surplus pepper generated by the pepper market during the harvest year can extend supply and stabilize the price of the pepper (41–43). Processed pepper products can also compensate fresh pepper shortages during lean and non-harvest years, thereby stabilizing the market.

In recent years, the Chinese government has been actively exploring the association between increasing the value of ecological products and increasing farmer income. The pepper industry has helped alleviate poverty and has realized higher returns for farmers. In Guizhou Province, pepper is an integral part of the agricultural industry (44). Pepper planting and processing have modulated the agricultural industry structure and increased revenue. Other management strategies for the improvement of pepper production include optimizing the layout of the industry according to regional climate and geography, constructing access roads and other transportation infrastructure, and enhancing the commercial value of agricultural products through effective packaging and marketing.

Croplands are artificial ecosystems based on natural ecosystem processes and occupy one-third of the Earth's land surface (45). Although croplands can also provide certain ecosystem services, compared with natural ecosystems that achieve internal circulation, croplands are characterized by relatively low species diversity and few simple interspecies relationships. Hence, the cropland ecosystem is relatively unstable and is subjected to various challenges (46). Nevertheless, croplands provide humans with food, fiber, and other products, and their wellbeing is essential for meeting basic human food requirements. Strategic agricultural design can increase the yield of the planting industry by enhancing agricultural production efficiency, limiting farmland expansion, and accommodating human food demands. The Chinese government has drawn a red line for basic cropland to guarantee a basic cropland supply. Reducing the demand for cropland areas within a certain range will provide more space for the implementation of several major national policies, such as reverting farmland to forest and grassland to restore and protect natural ecosystems. Sustainable food production and consumption promote resource recycling and help restore terrestrial ecosystems.

Pepper is produced mainly by open field cultivation and facility horticulture. The National Characteristic Vegetable Industry System reported that the planted area of open field cultivation accounts for 74% of the total. However, economically developed regions rely primarily upon facility gardening. The pepper planting areas and yield data evaluated in this study were segregated by cultivation method. We only analyzed the yield and area data for open field pepper cultivation to reveal ecological and geographic differences. Socioeconomic development factors have also strongly influenced the observed changes in pepper production and especially the rapid development of mechanized production and facility gardening. In future work, we will analyze the effects of these factors on pepper cultivation.

## Conclusion

The present study addressed the history of pepper introduction and distribution, identified the main pepper growing areas, and examined the regional differences in pepper consumption in China. We then analyzed and accounted for the regional differences in pepper production and consumption. Based on the foregoing content, we proposed suggestions for promoting the continued development of pepper cultivation and processing in China. The cost-effective and sustainable improvement of pepper production can help realize economic benefits, reduce the occupation of cultivated land, restore and conserve natural ecosystems, and establish and maintain human food security.

## Author Contributions

ZZ: formal analysis, data curation, and writing-original draft. XZ: supervision and funding acquisition. Both authors contributed to the article and approved the submitted version.

## Conflict of Interest

The authors declare that the research was conducted in the absence of any commercial or financial relationships that could be construed as a potential conflict of interest.

## Publisher's Note

All claims expressed in this article are solely those of the authors and do not necessarily represent those of their affiliated organizations, or those of the publisher, the editors and the reviewers. Any product that may be evaluated in this article, or claim that may be made by its manufacturer, is not guaranteed or endorsed by the publisher.
